# T1 and T2 mapping CMR to quantify focal myocardial injury in patients with myocarditis

**DOI:** 10.1186/1532-429X-17-S1-O90

**Published:** 2015-02-03

**Authors:** Ulf K Radunski, Sebastian Bohnen, Gunnar Lund, Dennis Säring, Christian Stehning, Bernhard Schnackenburg, Gerhard Adam, Stefan Blankenberg, Kai Muellerleile

**Affiliations:** 1 Radiology, University Medical Center Hamburg-Eppendorf, Hamburg, Germany; 2 Cardiology, University Heart Center Hamburg-Eppendorf, Hamburg, Germany; 3 Philips Healthcare, Hamburg, Germany; 4 Philips Research, Hamburg, Germany; 5 Computational Neuroscience, University Medical Center Hamburg-Eppendorf, Hamburg, Germany

## Background

Focal myocardial injury is an important diagnostic feature of myocarditis and is typically assessed by cardiovascular magnetic resonance (CMR) on late gadolinium enhancement (LGE) images. T1 mapping, T2 mapping, and extracellular volume (ECV) imaging are novel techniques which potentially improve the diagnostic value of CMR in myocarditis. This study evaluated the potential of T1 and T2 mapping CMR to assess focal myocardial lesions in myocarditis.

## Methods

We included 20 patients with myocarditis who had typical focal myocardial lesions on LGE images as reference method. Native T1, T2, and ECV maps were acquired in addition to a conventional CMR protocol at 1.5 Tesla. Myocardial lesions were quantified on LGE images by a standard threshold technique using a region of interest in normal appearing myocardium as reference tissue. Furthermore, myocardial lesions were quantified using normal values for native myocardial T1, T2, and ECV obtained from a group of 20 matched healthy controls as reference tissue. Injured myocardium was defined by a signal-intensity, native myocardial T1, T2 and ECV ≥ 2 standard deviations above reference values and expressed in percent of LV myocardium.

## Results

Median lesion size was 14% (9-20%) on LGE images. Areas with normal appearing myocardium on LGE images had significantly increased median native myocardial T1 and ECV values compared to myocardium of healthy volunteers (1085ms (1048-1120ms) vs. 1051ms (1021-1064ms); p<0.01 and 32% (30-35%) vs. 26% (24-27%; p<0.0001, respectively). Consequently, median lesion sizes were larger on native T1 maps (48% (32-56%); p<0.01) and ECV maps (58% (50-66%); p<0.01) compared to the median lesion size on LGE images. Median T2 values did not differ significantly between normal appearing myocardium of patients with myocarditis and myocardium of healthy volunteers (56 ms (54-60 ms) vs. 58 ms (53-62 ms); p=0.47). No significant difference in lesion size was found between T2 maps and LGE images (18% (9-38%); p=0.06).

## Conclusions

Native T1 and ECV maps reveal hidden myocardial injury in patients with myocarditis using myocardium of healthy controls as reference tissue.

## Funding

Dr. Marija Orlovic Foundation.

